# KAP1 Positively Modulates Influenza A Virus Replication by Interacting with PB2 and NS1 Proteins in Human Lung Epithelial Cells

**DOI:** 10.3390/v14040689

**Published:** 2022-03-26

**Authors:** Huapeng Feng, Ruonan Yi, Shixiang Wu, Genzhu Wang, Ruolin Sun, Liming Lin, Shunfan Zhu, Zhenyu Nie, Yulong He, Siquan Wang, Pei Wang, Jianhong Shu, Li Wu

**Affiliations:** 1 Department of Biopharmacy, College of Life Sciences and Medicine, Zhejiang Sci-Tech University, Hangzhou 310018, China; yirn1128@163.com (R.Y.); wsxzl0725@163.com (S.W.); wanggenzhu1232021@163.com (G.W.); srl991020@163.com (R.S.); llm980102@163.com (L.L.); zhushunfan2022@163.com (S.Z.); nzyzs1997@163.com (Z.N.); heyulong2003@163.com (Y.H.); w2584313659@163.com (S.W.); w2621373868@163.com (P.W.); 2 Department of Biology, College of Life Sciences, China Jiliang University, Hangzhou 310018, China

**Keywords:** KAP1, influenza A virus, interacting, PB2 and NS1, replication, human lung epithelial cells

## Abstract

Influenza virus only encodes a dozen of viral proteins, which need to use host machinery to complete the viral life cycle. Previously, KAP1 was identified as one host protein that potentially interacts with influenza viral proteins in HEK 293 cells. However, the role of KAP1 in influenza virus replication in human lung alveolar epithelial cells and the underlying mechanism remains unclear. In this study, we first generated KAP1 KO A549 cells by CRISPR/Cas9 gene editing. KAP1 deletion had no significant effect on the cell viability and lack of KAP1 expression significantly reduced the influenza A virus replication. Moreover, we demonstrated that KAP1 is involved in the influenza virus entry, transcription/replication of viral genome, and viral protein synthesis in human lung epithelial cells and confirmed that KAP1 interacted with PB2 and NS1 viral proteins during the virus infection. Further study showed that KAP1 inhibited the production of type I IFN and overexpression of KAP1 significantly reduced the IFN-β production. In addition, influenza virus infection induces the deSUMOylation and enhanced phosphorylation of KAP1. Our results suggested that KAP1 is required for the replication of influenza A virus and mediates the replication of influenza A virus by facilitating viral infectivity and synthesis of viral proteins, enhancing viral polymerase activity, and inhibiting the type I IFN production.

## 1. Introduction

Virus-host interactions play an important role in the viral life cycle. Influenza A virus encodes a limited number of proteins; it needs to make use of the host cellular machinery to complete its life cycle. Many host factors are involved in this process. These host proteins have been widely investigated by using genome-wide RNAi screen and CRISPR screen [1,2,3,4,5,6,7,8,9]. The mechanisms of action of most identified host proteins in the life cycle of the influenza virus were unknown. In the previous study, KRAB-associated protein 1 (KAP1), which is also known as Tripartite Motif Containing 28 (TRIM28), was shown to potentially interact with PB2, PB1, HA, NP, NA, and M1 proteins of influenza A virus by use of immunoprecipitations (IP) followed by mass spectrometry (MS) in HEK 293 cells [3]. In the other study, KAP1 was captured by the PR8-Flag-M2 and PR8-Flag-NS1 infection in the A549 cells (Heaton, N. et.al, *Immunity*, 2016). Bradel-Tretheway et al. reported that KAP1 may interact with the PA protein of the influenza virus in an RNA-dependent manner by comprehensive proteomic analysis [10]. Although KAP1 has been shown to be associated with the viral proteins of influenza virus, the specific viral proteins interacting with KAP1 have not yet been identified, and the mechanism of KAP1 mediating the replication of the influenza virus in A549 cells has still not been fully clarified.

KAP1 is composed of 835 amino acids including N-terminal RBCC-domain with E3 ubiquitin ligase activity possessed by most TRIM family members and a heterochromatin protein 1 binding domain (HP1 BD), a plant homeodomain (PHD), and a bromodomain (Bromo) at C-terminal [11,12]. KAP1 has been reported to be involved in the replication of several DNA viruses (e. g. Adenoviruses, Epstein-Barr virus) and RNA viruses (e.g., HIV, SARS-CoV-2) [13,14,15,16,17,18,19,20,21]. KAP1 inhibits the replication of human adenovirus (HdAV) by promoting the viral E1B-55K SUMOylation and restricts the HIV integration [17,20]. Furthermore, KAP1 is reported to inhibit the cell entry of SARS-CoV-2 by restricting the expression of the receptor ACE2 [21]. KAP1 has also been identified to be involved in the replication of the influenza A virus with genome-wide RNAi screen and system approaches [3,5,8,22]. KAP1 was also shown to be moderately up-regulated during influenza virus infection in A549 cells identified by quantitative phosphoproteomics analysis [23]. However, the role and mechanism of KAP1 in the influenza virus life cycle in the human lung epithelial cells (i.e., A549 cells) is not yet fully understood. In this study, we explored the role of KAP1 in the influenza life cycle in A549 cells. We found that KAP1 positively modulates the replication of influenza A virus by interacting with PB2 and NS1 proteins, and it mediates the replication of influenza A virus by facilitating the early step and synthesis of viral early proteins, enhancing viral polymerase activity, and inhibiting the type I IFN production.

## 2. Materials and Methods

### 2.1. Cells, Viruses and Ethics

Human lung epithelial cells A549 (Cat. No: CCL-185) purchased from the American Type Culture Collection (ATCC) were cultured in Ham’s F-12K medium (Wako) supplemented with 10% FBS and penicillin/streptomycin solution (Gibco). Madin–Darby Canine Kidney (MDCK) cells (Cat. No: CCL-34) were cultured in Eagle’s MEM (Gibco) supplemented with 5% NCS and penicillin/streptomycin solution.

Influenza A/WSN/33 virus (WSN; H1N1) was generated with reverse genetics as described previously [24] and propagated in MDCK cells. A/California/04/2009 (pdm H1N1) was propagated in MDCK cells. pPolI-WSN-PB2-KO/Rluc was constructed to replace the part of PB2 segment with the coding sequence of Renilla luciferase gene and keep the crucial region (120 nt) of packaging, termed as PB2(120) Rluc(120), and then the recombinant virus WSN-PB2-KO/Rluc was rescued by reverse genetics with plasmid pPolI-WSN-PB2-KO/Rluc, other seven-segment pPolI transcription plasmids and four expression plasmids(pCAGGS-WSN-PB2, pCAGGS-WSN-PB1, pCAGGS-WSN-PA, and pCAGGS-WSN-NP), as described previously [25], and was propagated in MDCK cells stably expressing the PB2 protein (MDCK/PB2). A/Puerto Rico/8/34 (H1N1; PR8)-NS1-R38AK41A virus was generated by using reverse genetics as described previously [24]. PR8-NS1-R38AK41A mutant virus was amplified in 10-day-old embryonic eggs and was stored at −70 °C until use. This study was approved by the Institutional Research Ethics Committee of the College of Life Sciences and Medicine, Zhejiang Sci-Tech University.

### 2.2. siRNA Transfection and Knock-Down Efficacy Detection

A549 cells were transfected with two siRNAs targeting Trim28 including Hs_TRIM28_6 FlexiTube siRNA and Hs_TRIM28_8 FlexiTube siRNA purchased from Qiagen (named as siKAP1-1# and siKAP1-2#) by using RNAiMAX transfection reagent (Thermo Fisher Scientific, Waltham, MA, USA). Allstars negative control siRNA (Qiagen) was used as a negative control, siRNA targeting the nucleoprotein (NP) gene of influenza A virus (siNP: GGA UCU UAU UUC UUC GGA GUU) as the positive control. At 48 h post-transfection, part of the cells was harvested for knockdown efficacy detection by using qRT-PCR and Western blotting.

### 2.3. Generation of the KAP1 Overexpression and Knockout A549 Cells

The open reading frame of human Kap1 gene was amplified from the cDNA of A549 cells and added Flag tag at C-terminal by primers and cloned into the multiple cloning site (MCS) A of the expression vector pIRES-puromycin which carries the puromycin resistant gene in MCS B site. And the plasmids pIRES-KAP1-Flag and pIRES-puromycin were transfected into the A549 cells with the Lipofectamine^®^ LTX & Plus Reagent (Thermo Fisher Scientific, Waltham, MA, USA) according to the manufacturer’s instruction. At 24 h post-transfection, 1 µg/mL puromycin was added into the medium of selection, the cell clones were picked up after 14 days selection. Two aliquots were seeded for each clone; one aliquot is for Western blotting detection and the other is for amplification as seeds.

The guide RNAs targeting the human *Kap1* gene were designed by use of the website software (http://crispr.mit.edu/). The sequence of single-guide RNA is as follows: sense oligo CACCGGAGCGCTTTTCGCCGCCAG; antisense oligo AAACCTGGCGGCGAAAAGCGCTCC. The oligos were phosphorylated, annealed, and cloned into the BbsI-digested pX330 vector (Addgene). And the pX330-KAP1-sgRNA plasmid was confirmed by using Sanger sequencing. The plasmids pX330-KAP1-sgRNA were transfected into the A549 cells with the Lipofectamine^®^ LTX & Plus Reagent (Thermo Fisher Scientific, Waltham, MA, USA). At 24 h post-transfection, the cells were plated into 96-well plates, 0.5 cells per well. The cells were maintained for three weeks and the cell clones were picked up into 24-well plates, two aliquots per clone. And ten days later, one aliquot was used to identify the absence of KAP1 protein expression with sequencing and Western blotting, the other corresponding one was used to amplify and make the cell stocks.

### 2.4. Immunoprecipitation

The KAP1 overexpression A549 cells were plated into the 10 cm^2^ dish. After 24 h, the cells were inoculated with the WSN virus at an MOI of 10. At 12 h post- infection, the cells were harvested and lysed in IP lysis buffer (50 mM Tris-HCl with pH 7.5, 150 mM NaCl, 1 mM EDTA, 0.5% Nonidet P-40) for 30 min at 4 °C with protease inhibitor mixture complete mini (Roche). The precleared lysates were aliquoted, and one was incubated with 1 µg mouse normal IgG and the other one was incubated with 1 µg mouse anti-Flag antibody (F1084, Sigma) for 3 h. An amount of 50 µL of Dynabeads (Invitrogen) was added and further incubated for 16 h at 4 °C. The beads were washed with lysis buffer four times and the immunoprecipitation complex was eluted with 2 × SDS sample buffer for 10 min at 70 °C and then DTT was added to the elution for Western blotting.

### 2.5. Antibodies and Western Blotting

Primary antibodies used in this study include rabbit anti-KAP1 antibody (ab10484; Abcam), mouse anti-KAP1 antibody (ab22553; Abcam), rabbit anti-Phospho KAP1 (S824) antibody (A300-767A, Bethyl); mouse anti-β-actin antibody (A2228; Sigma-Aldrich, Darmstadt, Germany); mouse anti-FLAG antibody (F1084, Sigma-Aldrich), rabbit anti-influenza A NS1 polyclonal antibody purchased from GeneTex, Inc. (GTX125990 Q-1; Irvine, CA, USA). Mouse anti-M1antibody and mouse anti-HA antibody, mouse anti-NP, rabbit anti-NS2, rabbit anti-PB2, rabbit anti-PB1, rabbit anti-PA, mouse anti-M2, and rabbit anti-WSN virus antibodies were available in our laboratory. Secondary antibodies were used as follows: HRP-conjugated sheep anti-mouse IgG (GE Healthcare, NA931), HRP-conjugated donkey anti-rabbit IgG (GE Healthcare, NA934), HRP-conjugated Clean-blot IP detection reagent (Thermo Fisher Scientific, Waltham, MA, USA, 21230). HRP-conjugated rat monoclonal (H139-52.1] anti-mouse kappa light chain (Abcam, ab99632) and HRP conjugated anti-mouse IgG (Abcam, ab131368) secondary antibodies for IP blot.

### 2.6. Virus Growth Kinetics

Wild-type and KAP1 KO A549 cells were plated into the six-well plates and were infected with A/WSN/33 virus (WSN; H1N1), A/California/04/2009 (H1N1 pdm) at an MOI of 0.001, respectively. The cells infected with the WSN virus were cultured in the Ham’s F12K with 0.3% BSA and 0.1% FCS. The cells infected with the other two influenza viruses were maintained in the Ham’s F12K with 0.3% BSA and 0.4 µg/mL L-(tosylamido-2-phenyl) ethyl chloromethyl ketone (TPCK)-treated trypsin. The infected cells were incubated at 33 °C (pdm H1N1 virus) or 37 °C (WSN virus). The supernatants were collected at various time points (12 h, 24 h, 36 h, 48 h, 60 h, and 72 h). The viral titers were determined by using plaque assay in MDCK cells. 

### 2.7. Immunofluorescence

The A549 cells were seeded into glass-bottom dishes and were infected with the WSN virus at an MOI of 10. At an indicated time, post-infection, the cells were washed with cold PBS and fixed with 4% paraformaldehyde for 30 min at room temperature or 4 °C overnight. The cells were washed twice and were penetrated with 0.1% Triton X-100 for 15 min at room temperature. Then the cells were blocked with Blocking One for 1h at room temperature. The primary antibodies were diluted in the Blocking One and were incubated at 4 °C overnight. After four times wash, the Alexa Fluor 488 or Alexa Fluor 594 secondary antibodies diluted in 20% Blocking one in PBS-T with Hoechst 33342 (Invitrogen, 10,000×) were incubated for 1 h at room temperature. The cells were washed four times and were observed with a Zeiss LSM 780 Confocal Microscope.

### 2.8. PB2-KO/Rluc Virus Assay

KAP1 KO and wild-type A549 cells were plated into the 24-well plate. After 24 h, the cells were infected with WSN PB2-KO/Rluc virus at an MOI of 1. An amount of 100 μg/mL amantadine (Sigma) was used as a positive control. After 1 h incubation, the cells were washed and the Ham’s F12K with 0.3% BSA with or without amantadine was added. At 8 h post-infection, the supernatants were removed, and the attached cells were subjected to detection by use of a *Renilla* Luciferase Assay System (Promega) with a GloMAX Navigator (Promega) according to the manufacturer’s instructions.

### 2.9. Mini-Genome Assay

KAP1 KO and wild-type A549 cells were plated into a 24-well plate. After 24 h, the plasmids pCAGGS expression PB2, PB1, PA, and NP were transfected by using Lipofectamine^®^ LTX & Plus Reagent (Thermo Fisher Scientific, Waltham, MA, USA) together with a plasmid expressing luciferase from a virus-like RNA encoding firefly luciferase (pPolINP(0)luc2(0)) and pNull renilla luciferase (pNull-Rluc) as a transfection efficiency control [26]. After 24 h post-infection, the transfected cells were lysed and the firefly luciferase activity and *Renilla* luciferase activity were measured with Dual-Glo Luciferase Assay System (Promega, E2920).

### 2.10. IFN-β ELISA

WT and KAP1 overexpression A549 cells were infected with PR8-NS1-R38AK41A virus at an MOI of 1 and incubated for 24 h after infection at 37 °C. The supernatants were collected and interferon-β was measured by using a VeriKine Human Interferon Beta ELISA Kit (PBL Assay Science, NJ, USA) according to the manufacturer’s instructions. ELISA plates were detected at an absorbance of 450 nm by using a Versa Max plate reader (Molecular Devices, San Jose, CA, USA).

### 2.11. VLP Formation Assay

The KAP1 KO and wild-type A549 cells were transfected with pCAGGS plasmids expressing HA, NA, and M1 of WSN virus with Lipofectamine^®^ LTX & Plus Reagent (Thermo Fisher Scientific, Waltham, MA, USA). After 48 h, the supernatants were collected and subjected to centrifugation at 3000 g for 10 min at 4 °C. Then 3.3 mL of PBS was added into the ultracentrifuge tube using a pipette and the above-precleared supernatants were transferred into the tube and mixed. An amount of 0.7 mL 30% sucrose at the bottom was added, followed by ultracentrifugation at 40,000 rpm for 2 h at 4 °C. After ultracentrifugation, 50 µL 2 × SDS sample buffer with dithiothreitol (DTT) was added. For the transfected cells, we added 250 µL 2 × SDS sample buffer with DTT into each well, sonicated them for 10 min, and heated them at 95 °C for 10 min for Western blotting.

### 2.12. Single-Molecule Fluorescence In Situ Hybridization (smFISH)

The KAP1 KO and wild-type A549 cells were plated onto poly-lysine coated cover glasses (Corning) at a density of 1 × 10^5^ cells/well and grown overnight at 37 °C. The cells were infected with the WSN virus at an MOI of 10. At 12 h post-infection, cells were washed once with cold PBS, followed by fixation with 4% paraformaldehyde in PBS for 30 min at RT. After washing with cold PBS, the cells were permeabilized with 0.5% Triton X-100 in PBS-T for 15 min at RT. The cells were then washed with PBS and incubated in 2 × SSC (300 mM sodium chloride, 30 mM sodium citrate) with 10% formamide for 5 min before hybridization. To detect viral RNAs, 4 mM of labeled probes detecting segment 7 of influenza virus in 40 mL of hybridization buffer (10% dextran sulfate, 2 mM vanadyl ribonucleoside complexes (VRC, New England BioLabs), 0.02% RNAse free BSA, 50 mg *E. coli* tRNA, 2 × SSC, 10% formamide) was used for each sample. Hybridization was carried out in humidified chambers maintained at 37 °C for 16 h. The samples were then washed twice with 10% formamide 2 × SSC supplemented with 2 mM VRC for 30 min at 37 °C. Nuclear staining was performed by using 0.5 mg/mL Hoechst 33342 and the coverslips were mounted in ProLong Gold anti-fade mounting media (Invitrogen). The cured samples were subjected to confocal microscopy examination.

### 2.13. Statistical Analysis

Statistical analysis (one-way or two-way analysis of variance [ANOVA] followed by Dunnett’s test) was performed by using GraphPad Prism 6.07. *p* < 0.05 was considered to indicate a statistically significant difference. 

## 3. Results

### 3.1. KAP1 Is Required for Influenza A Replication in A549 Cells

**Figure 1:**
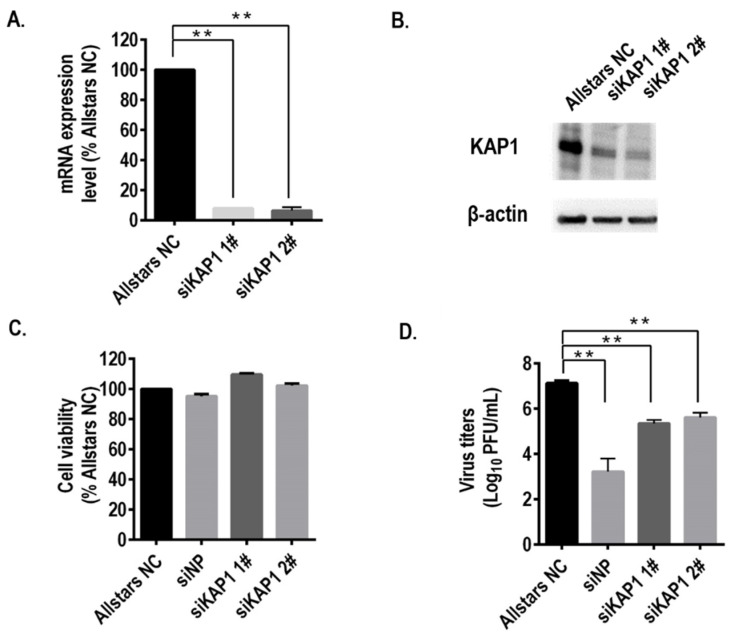
Knockdown of KAP1 resulted in a reduction in influenza virus replication. A549 cells were transfected twice with negative control siRNA (Allstars NC) or two siRNAs targeting human Kap1 (siKAP1 1#; siKAP1 2#). (**A**) qPCR to confirm the relatively low level of mRNA expression in siKAP1-treated A549 cells. (**B**) Western blotting with anti-KAP1 or anti-β-actin antibody to confirm the knockdown efficiency. (**C**) Cell viability of siRNA-treated cells was determined by using CellTiter-Glo reagent. (**D**) Virus growth in siRNA-treated A549 cells infected with WSN virus at an MOI of 0.001 and the supernatant was collected at 48h post-infection. (h.p.i: hours post-infection). Means ± SD of triplicate experiments is shown in (**A**,**C**,**D**). ** *p* < 0.01 (two-way ANOVA followed by Dunnett’s test).

To examine whether KAP1 plays a role in the replication of the influenza A virus in human lung epithelial cells, we transfected two KAP1-specific siRNAs into A549 cells and the efficiency of knockdown of KAP1 was confirmed with qPCR and Western blotting (Figure 1A, B). The viability of the cells treated with KAP1 siRNAs and control siRNA (Allstars NC) were comparable (Figure 1C). The cells transfected with KAP1 siRNAs and control siRNAs were infected with the WSN virus at a multiplicity of infection (MOI) of 0.001. Virus titers in KAP1 knockdown cells were significantly lower than those in negative control siRNA treated cells (Figure 1D), this indicates that KAP1 is required for the influenza virus life cycle.

**Figure 2:**
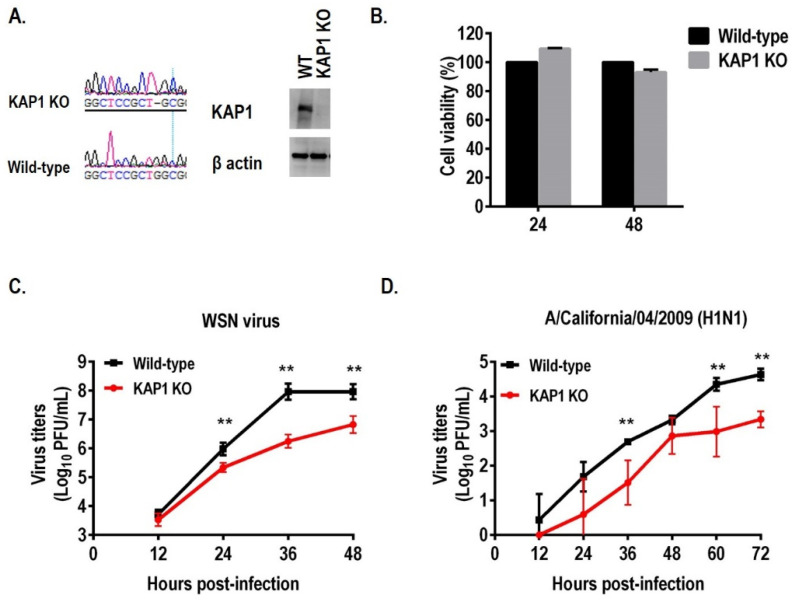
Knockout of KAP1 in A549 cells significantly reduced the influenza A virus growth. (**A**) One nucleotide “G” at position 82 of the KAP1 mRNA was deleted, this was confirmed by Sanger sequencing, and the absence of KAP1 expression in A549 cells was validated by Western blotting. (**B**) Cell viability of KAP1 was detected by using CellTiter-Glo reagent. (**C**) Deletion of KAP1 resulted in a significant reduction of WSN virus growth. (**D**) Deletion of KAP1 resulted in the significant reduction of CA04 pdmH1N1 virus growth. ** *p* < 0.01 (two-way ANOVA followed by Dunnett’s test).

To further confirm the role of KAP1 protein in the replication of influenza virus in A549 cells, we generated the KAP1 knockout (KO) A549 cells by using the CRISPR/Cas9 system for gene editing. Wild-type A549 cells (WT) were transfected with pX330 encoding gRNA1, targeting exon 1 of the KAP1 gene. The clones were picked up and sequenced, and we found that one nucleotide “G” at position 82 of the Kap1 mRNA was deleted in clone 3 and resulted in a frameshift and the stop codon appears at position 91 (Figure 2A). The absence of KAP1 protein in the KAP1 KO A549 cell clone was validated by Western blotting with an anti-KAP1 antibody (Figure 2A). In addition, the viability of KAP1 KO cells was similar to that of WT A549 cells at 24 h after seeding, which was the time point at which we infected the cells with the virus (Figure 2B). To investigate the virus growth dynamics in the KAP1 KO A549 cells, the WT and KAP1 KO cells were infected with WSN virus at an MOI of 0.001 for detecting the multiple cycles of viral replication, the supernatants were harvested at the indicated time points, and virus titers were determined by using a plaque assay. Depletion of KAP1 protein significantly reduced the WSN virus titers up to 1.6 log10 units (Figure 2C). In addition, deletion of KAP1 A549 cells resulted in the significant reduction of virus titers of other influenza virus strains A/California/04/2009 (pdm H1N1) (Figure 2D). To further confirm this result, we generated another KAP1 KO A549 cell clone-clone23 and the gene editing pattern is different from the clone 3, the result showed that deletion of KAP1 also significantly decreased the influenza virus growth (Figure S1). These data demonstrate that KAP1 promotes the replication of the influenza virus in A549 cells.

### 3.2. Overexpression of KAP1 Promotes the Replication of Influenza Virus in A549 Cells

**Figure 3:**
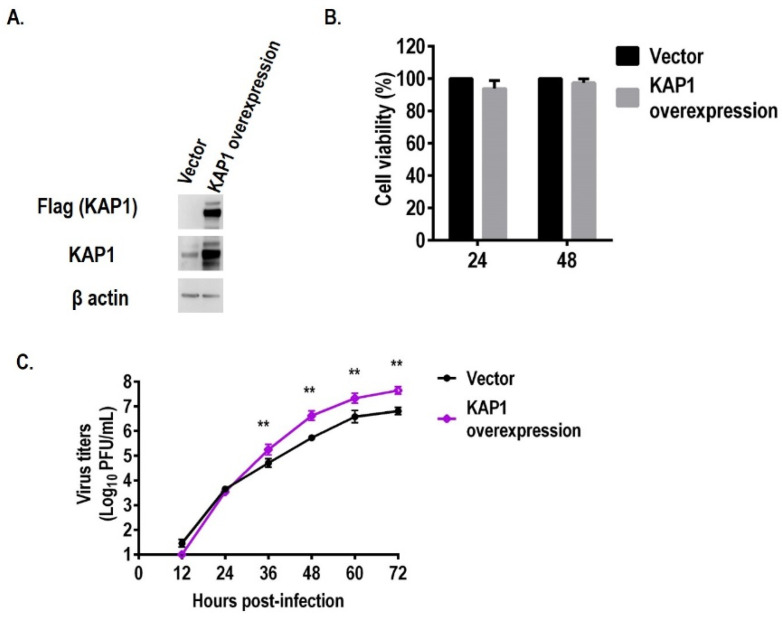
Overexpression of KAP1 in A549 cells enhanced the influenza A virus replication. (**A**) Overexpression of KAP1 in A549 cells was confirmed with anti-FLAG and anti-KAP1 antibodies. (**B**) Cell viability of KAP1 overexpressed A549 cells determined by CellTier-Glo reagent. (**C**) Overexpression of KAP1 in A549 cells enhanced the WSN virus growth. ** *p* < 0.01 (two-way ANOVA followed by Dunnett’s test).

To further confirm the above results, we constructed the KAP1 stably expression A549 cells. The expression level of KAP1 in A549 cells was higher than that in vector-control A549 cells confirmed by Western blotting (Figure 3A). The virus titers in KAP1-overexpression A549 cells were also higher than that in vector-control A549 cells at 36, 48, 60, and 72 hpi (e.g., 10^5.24^ pfu/mL *VS* 10^4.52^ pfu/mL at 36 hpi) while the cell viability of these two kinds of A549 cells were similar (Figure 3B,C). These results demonstrate that KAP1 is a pro-viral host factor of influenza A virus in A549 cells.

### 3.3. KAP1 Interacted with PB2 and NS1 Proteins

**Figure 4:**
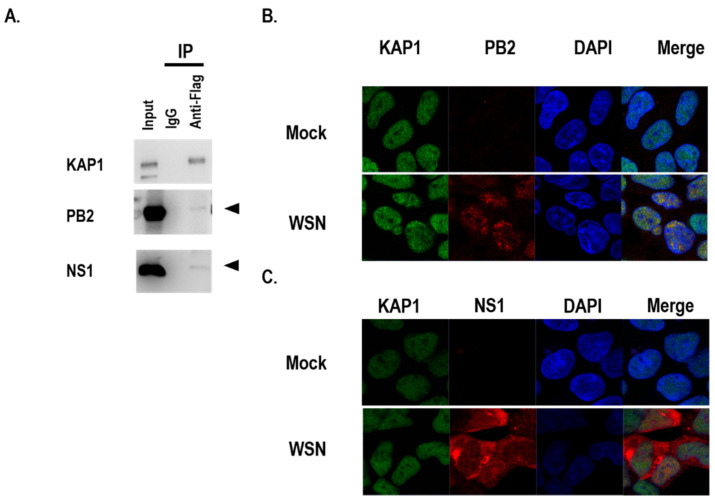
KAP1 interacted with PB2 and NS1 proteins during influenza A virus infection in A549 cells. (**A**) WT or KAP1-FLAG overexpression A549 cells were infected with the WSN virus at an MOI of 10. The cell lysates were harvested at 12 hpi and immunoprecipitated (IP) by using an anti-FLAG antibody and Dynabeads. The products were subjected to Western blotting. (**B**) A549 cells were infected with the WSN virus at an MOI of 10. The cells were fixed and permeabilized at 8 h post-infection and then immunostained with anti-PB2, and anti-KAP1 antibodies and then were observed using a confocal microscopy. (**C**) A549 cells were infected with the WSN virus at an MOI of 10. The cells were fixed and permeabilized at 8 h post-infection and then immunostained with anti-NS1, and anti-KAP1 antibodies and then were observed using a confocal microscopy.

In the previous study, KAP1 was co-precipitated with PB2, PB1, HA, NP, NA, and M1 proteins with Flag tag by transfection and IP-MS in HEK 293 cells [3]. Heaton, et al. identified that KAP1 was co-precipitated with M2 and NS1 Flag-tagged influenza viruses by infection and IP-MS in A549 cells [22]. To confirm which viral proteins interacted with KAP1 in A549 cells during influenza virus infection, we first established the KAP1-Flag stable overexpression A549 cells and then examined the interaction between KAP1 and several viral proteins including PB2, PB1, PA, HA, NP, M1, M2, and NS1 in the KAP1 stable overexpression A549 cells infected with WSN virus. We found that PB2 and NS1 were co-immunoprecipitated with KAP1 (Figure 4A and Figure S2). To examine KAP1-PB2 and KAP1-NS1 co-localization during the virus infection, A549 cells were infected with the WSN virus at an MOI of 10, the cells were fixed at 8 h post-infection. KAP1 protein localized in the nucleus in mock- and WSN virus-infected A549 cells. The PB2 proteins are mainly localized in the nucleus and co-localized with KAP1 in the nucleus (Figure 4B). NS1 protein is distributed in the cytoplasm and nucleus and partially co-localized with KAP1 in the nucleus (Figure 4C). These data indicated that influenza A virus infection did not change the distribution of KAP1 and KAP1 may play its role in the influenza virus life cycle by interacting with PB2 and NS1 proteins.

### 3.4. KAP1 Is Involved in the Early Steps of Influenza Virus Replication

**Figure 5:**
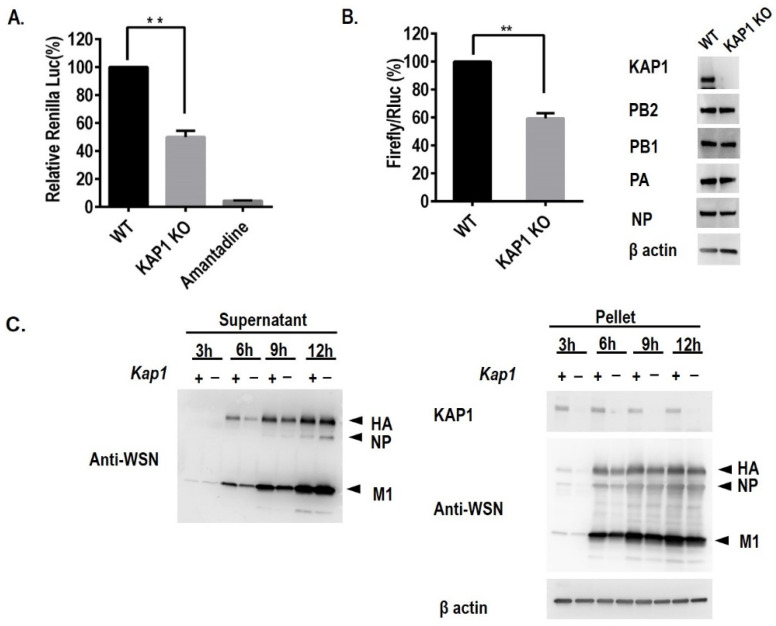
Role of KAP1 in the multiple steps of the influenza virus life cycle. (**A**) Role of KAP1 in the early steps of influenza virus replication. WT and KAP1 KO A549 cells were infected with PB2-KO/Rluc virus at an MOI of 1. Amantadine was used as a positive control. Renilla luciferase activity was measured at 8 h post-infection. Values represent the means of triplicate experiments ± SD. ** *p* < 0.01 (one-way ANOVA followed by Dunnett’s test). (**B**) Role of KAP1 in the mini-genome replicon. WT and KAP1 KO A549 cells were transfected in triplicate with a luciferase reporter plasmid and an internal control plasmid, together with plasmids expressing PB1, PB2, PA, and NP from WSN virus, the firefly luciferase and Renilla luciferase activity were measured with the Dual-Glo luciferase assay system. The values shown are means ± standard deviations of data from triplicate experiments and are normalized to the activity of that in WT cells (100%). **, *p* < 0.01 compared with that in WT A549 cells. Right panel: Plasmid-transfected A549 cells were homogenized in a 2 × SDS sample buffer and then analyzed by Western blotting to measure the expression levels of PB2, PB1, PA, and NP. (**C**) Role of KAP1 in viral protein expression. The WT and KAP1 KO A549 cells were infected with WSN at an MOI of 10. At 3, 6, 9, and 12 hpi, total cell lysates were analyzed by Western blotting with anti-KAP1 antibody and anti-WSN virus antibody (R309, for detecting HA, NP, and M1 proteins); β-actin served as a loading control.

To examine whether KAP1 is involved in the early step of influenza virus replication, we used a replication-incompetent PB2-knockout WSN virus (PB2-KO/Rluc virus), whose PB2 gene has been replaced with that of the Renilla luciferase reporter protein, as described previously [25]. As this virus lacks a functional PB2 gene, Renilla luciferase expression is indicative of viral infectivity. WT and KAP1 KO A549 cells were infected with PB2-KO/Rluc virus at an MOI of 1 to make sure most of the cells were infected and reflect the effect on the early steps in viral replication in one viral life cycles. Amantadine was used as a positive control because it inhibits viral HA-mediated membrane fusion [27]. Renilla luciferase activity was measured at 8 h post-infection (hpi). Lack of KAP1 expression and amantadine treatment resulted in a significant reduction of Renilla luciferase activity (Figure 5A). The activity of Renilla luciferase activity in KAP1 KO A549 cells was reduced by 50–60% compared with that in WT A549 cells. This result demonstrated that KAP1 positively modulates the early steps of the influenza virus life cycle.

### 3.5. Lack of KAP1 Reduced the Polymerase Activity of the Influenza A Virus

Based on the interaction between KAP1 and PB2, one important component of the polymerase complex of the influenza virus, we speculated that KAP1 may be associated with the polymerase activity of the influenza virus. To confirm this, WT A549 and KAP1 KO cells were transfected with plasmids expression the PB2, PB1, PA, and NP protein of influenza A virus and the reporter plasmid pPolINP(0)luc2(0) and internal control plasmid pNull-Rluc. We measured the activity of reporter and internal luciferase activity and compared them to indicate the polymerase activity. We found that deletion of KAP1 significantly reduced the polymerase activity (Figure 5B). Deletion of KAP1 did not significantly alter the expression level of four viral proteins (PB2, PB1, PA, and NP) in A549 cells by transfection (Figure 5B). This result indicates that KAP1 is required for maintaining the polymerase activity of the influenza virus in A549 cells.

### 3.6. KAP1 Is Necessary for the Expression of Viral Proteins

Previous studies have demonstrated that KAP1 is a novel transcriptional elongation factor [28,29]. To evaluate whether KAP1 affects the expression of the viral proteins, WT and KAP1 KO A549 cells were infected with the WSN virus at an MOI of 10 to make sure every cell was infected, and the sufficient viral proteins were synthesized for detection in one viral life cycle; the supernatant and total cell lysate were prepared at 3, 6, 9, and 12 hpi, respectively. The virus particles in the supernatant were ultra-centrifugated. After 6 hpi, the expression of HA in the supernatant of infected KAP1 KO cells was lower than that of wild-type A549 cells, and at 6 and 9 hpi, expression of M1 in the supernatant of infected KAP1 KO cells was lower than that of wild-type A549 cells (Figure 5C). In the cell lysate, expression of HA and NP in KAP1 KO cells were less than that in WT A549 cells at 3 and 6 hpi. While the expression of M1 decreased following the deletion of KAP1 in A549 cells at 6 hpi (Figure 5C). Taken together, these data indicate that KAP1 plays an important role in the expression of viral proteins. In addition, we found that the deletion of KAP1 did not affect the VLP formation and release of influenza virus by transfection and M1 vRNA nuclear translocation (Figures S3 and S4).

### 3.7. KAP1 Negatively Regulated the Interferon Production after Influenza Virus Infection

**Figure 6:**
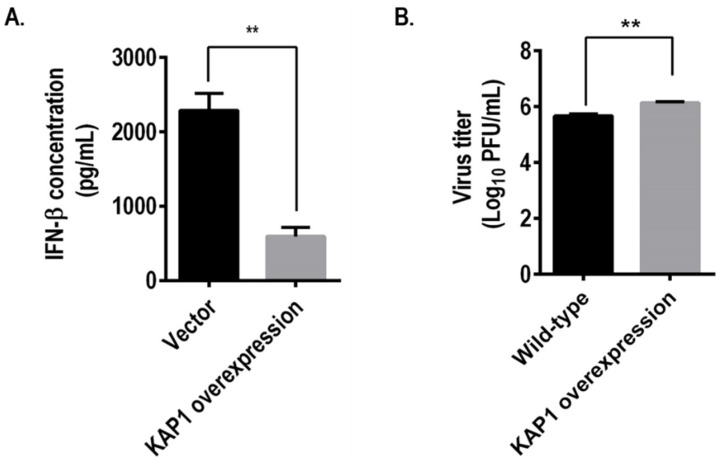
Role of KAP1 in the IFN-β production induced by influenza virus infection. (**A**) WT and KAP1 overexpression A549 cells were infected with PR8-NS1-R38AK41A virus at an MOI of 1 and incubated for 24 h after infection at 37 °C. The supernatants were collected and interferon-β was measured by using a VeriKine Human Interferon Beta ELISA Kit. (**B**) Virus titers of PR8-NS1-R38AK41A in the supernatant were determined by using a plaque assay. ** *p* < 0.01 (one-way ANOVA followed by Dunnett’s test).

Many TRIM family members have been reported to be involved in interferon production [30,31,32,33,34]. Our precipitation experiment has shown that KAP1 interacted with NS1 protein (Figure 4), which has been demonstrated to inhibit interferon production during the infection of influenza virus [35,36]. This information prompted us to examine whether KAP1 is associated with the interferon production during influenza virus replication. To this end, we infected the vector control and KAP1 overexpression A549 cells with A/Puerto Rico/8/1934-NS1-R38AK41A (H1N1) virus at an MOI of 1, respectively, which has been reported to induce the production of the interferon previously [37,38]. And measured the amount of interferon in the medium of infected cells at 24 hpi. The result showed that the amount of interferon β was significantly decreased in the supernatant of infected KAP1 stably expression A549 cells (Figure 6A). The virus titers in the supernatant of infected KAP1 stably expression A549 cells were higher than that in vector control cells (Figure 6B). These data demonstrate that KAP1 inhibits the production of interferon during the infection of the influenza A virus.

### 3.8. KAP1 Was deSUMOylated and Phosphorylated during Influenza Infection

**Figure 7:**
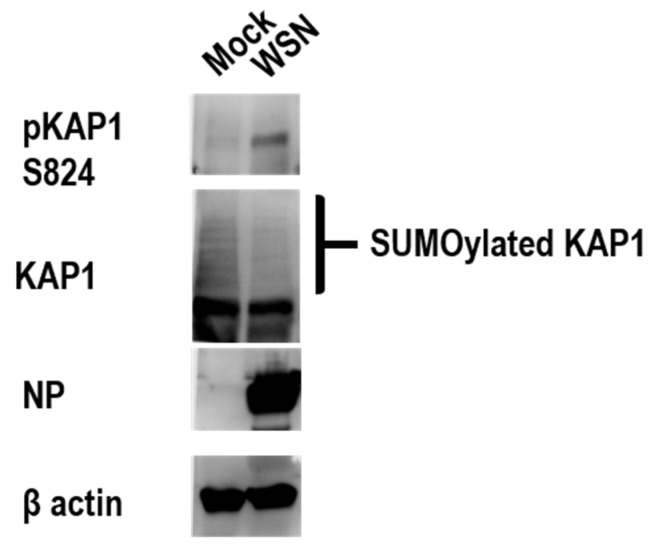
Influenza A virus induced the deSUMOylation and phosphorylation of KAP1 in A549 cells. A549 cells were infected with WSN virus at an MOI of 10, the infected cells were harvested at 12 hpi and suspended in a 2 × SDS sample buffer and then were subjected to Western blotting for analysis with the indicated antibodies.

A previous study reported that KAP1 was phosphorylated during the adenovirus infection in H1299 and A549 cells [17]. So we wondered whether KAP1 was phosphorylated during the influenza virus in A549 cells. To examine this, WT A549 cells were infected with the WSN virus at an MOI of 10 to ensure that the virus can infect every cell and further affect the posttranslational modification of KAP1 during the one viral life cycle, and the cells were harvested at 12 h post-infection and subjected to Western blotting. The result showed that the total KAP1 protein was comparable between mock and virus-infected A549 cells; in contrast, the phosphorylated KAP1 was increased after WSN virus infection (Figure 7). At the same time, we found that the SUMOylation of KAP1 was decreased after WSN virus infection (Figure 7). This indicated that the influenza infection induced the KAP1 deSUMOylation and phosphorylation switch.

## 4. Discussion

Viral-host interaction plays an important role in the influenza virus life cycle. KAP1 was identified as one of the host proteins potentially interacting with several viral proteins in the previous study in HEK 293 cells and was reported to potentially interact with NS1 and M2 proteins [3,22]. However, the role of KAP1 in the influenza virus life cycle in the human lung epithelial cells is not fully clear. In this study, we found that KAP1 promotes the replication of the influenza A virus by using deletion and overexpression of KAP1 proteins in A549 cells. Further studies demonstrated that KAP1 is involved in multiple steps of the influenza life cycle including early steps in viral replication, transcription and replication of viral genome, and viral protein synthesis.

Many TRIM family members restrict viral growth and some of them play pro-viral replication [32]. Several TRIM family members have been reported to be involved in the replication of influenza viruses including TRIM22, TRIM25, TRIM29, TRIM32, TRIM35, TRIM41, and TRIM56 [39,40,41,42,43,44,45,46]. TRIM22 restricts the influenza A virus replication by degrading the viral nucleoprotein [43]. TRIM25 targeted the influenza virus ribonucleoproteins to block viral RNA synthesis [40]. TRIM32 inhibits the growth of the influenza virus through ubiquitination of PB1 in mammals and restricted the H5N6 virus replication through mediating the IFN production in ducks [42,45]. TRIM56 C-terminal tail impedes the replication of influenza A and B viruses by restricting the viral RNA synthesis [41]. Recently, TRIM35 was demonstrated to restrict influenza virus replication by activating TRAF3 and promoting the degradation of viral PB2 protein [46]. TRIM41 was shown to limit influenza A virus infection by targeting NP for ubiquitination and protein degradation [39]. In addition, TRIM29 was found to inhibit the production of type I interferons in macrophage, and the deletion of TRIM29 can protect mice from infection with influenza virus, these results indicate that TRIM29 promotes the in vitro and in vivo growth of the influenza virus [47]. In this study, we found KAP1 is required for the influenza A virus replication in A549 cells (Figure 1D and Figure 2C,D). Further study is needed to explore why KAP1 plays different role from other TRIM family members (e.g., TRIM22, TRIM32, TRIM41, TRIM56) during the influenza virus infection although all of them contain RING domain, B-Box domain, and Coiled-coil domain. As a previous study reported [41], C-Terminus of these TRIM family members may play an important role in the replication of the influenza virus, so we speculate that KAP1 C-terminus may determine its different role from other TRIM family members (e.g., TRIM22, TRIM32, TRIM41, TRIM56) in the influenza virus life cycle.

The influenza virus life cycle can be divided into five stages including viral entry into host cells, importing vRNP into the nucleus, transcription and replication of the viral genome, nuclear export of vRNP, and assembly and budding. In this study, we designed specific experiments to systematically investigate the role of KAP1 in the influenza viral life cycle. We found that KAP1 is involved in viral infectivity, viral protein synthesis, and the transcription and replication of viral genome of influenza virus life cycle (Figure 5A–C). Deletion of KAP1 did not significantly affect the import/export of M vRNA, and VLP formation and release (Figures S3 and S4), this indicates that KAP1 is not involved in the nuclear transport of vRNP and assembly and budding. Recently, some researchers found that TRIM28 restricts viral entry of SRAS-CoV-2 by mediating the expression of ACE2 [21]; however, in this study, we found that KAP1 facilitates the viral infectivity of influenza virus, this different role may be due to the different receptors or mechanisms of viral replication (Figure 5A). In addition, we also found that the deletion of KAP1 resulted in a significant decrease in polymerase activity. Bunch et al. reported that KAP1 is a novel transcriptional elongation factor and it can regulate RNA polymerase II promoter-proximal pausing and pause release [28,29]. In this study, we found that KAP1 interacted with PB2 protein, one important subunit of influenza virus polymerase, and the deletion of KAP1 resulted in the reduction of the *Rinella* luciferase activity in the PB2-KO/Rluc virus assay, reduction of Luciferase activity in mini-genome assay, and viral protein synthesis (Figure 5). We speculated that these effects may result from the interaction between KAP1 and PB2, one subunit of viral polymerase, and further resulted in the reduction of vRNA synthesis (Figure 4 and Figure 5B). Although we still observed the significant decrease of activity of *Renilla* luciferase by using WSN-PB2 KO/Rluc virus infection without PB2, this demonstrates that KAP1-PB2 interaction may just play a role in the influenza life cycle and KAP1 affects the viral replication through multiple aspects such as innate immune response and posttranslational modification as shown in Figure 6 and Figure 7. As shown in Figure 4, KAP1 interacted with NS1 protein, which is an antagonist of the production of type I IFN [48,49]. And we found that overexpression KAP1 resulted in a significant decrease of the type I interferon production induced by influenza A virus infection. Some other host proteins have also been found to interact with more than one viral protein of the influenza virus and exert different functions in the influenza virus life cycle. Chen et al. revealed that DDX21 interacted with PB1 to inhibit the viral RNA and protein synthesis, but influenza A virus likely uses the DDX21-NS1 interaction to overcome restriction to regulate the viral life cycle [50]. TRIM25 was first demonstrated to be targeted by NS1 of influenza virus to inhibit the host antiviral response and then the other group further found TRIM25 can bind vRNP to restrict the viral RNA synthesis [40,44]. In this study, we found that KAP1 interacted with PB2 and NS1 proteins during influenza virus infection, and KAP1 is required for influenza virus replication. We speculated that KAP1-PB2 interaction may facilitate the viral RNA and protein synthesis and KAP1-NS1 may be associated with the host antiviral response. During our ongoing study, two other groups reported that KAP1 mediates the replication of the influenza virus by inducing the antiviral innate immune responses [51,52]. Therefore, we demonstrated that KAP1 positively modulates the propagation of the influenza A virus by interacting with the PB2 and NS1 proteins in human lung epithelial cells. Previously, Bürck et al. showed that human adenoviruses induced deSUMOylation and phosphorylation of KAP1 in the adenovirus-infected cells and promoted SUMOylation of viral protein E1B-55K [17]. In this study, we found that the infection of the influenza A virus induced the KAP1 deSUMOylation and phosphorylation switch, and KAP1 interacted with NS1 of the influenza virus. A previous study has demonstrated that the SUMOylation enhances NS1 stability and thus promotes rapid growth of the influenza A virus [53,54]. So KAP1 may induce SUMOylation of the viral protein NS1 and therefore plays a major pro-viral role during influenza A virus infection.

In summary, our results reveal that KAP1 is required for influenza A virus infection and is involved in several steps in the influenza life cycle including promoting viral infectivity, facilitating the viral protein synthesis, and maintaining the polymerase activity in human lung epithelial cells. In addition, we also found that KAP1 restricts the IFN-I production, possibly through interacting with NS1 protein. Furthermore, influenza A virus infection induced the KAP1 deSUMOylation and phosphorylation switch, which also can enhance the viral gene expression to facilitate the virus propagation. A detailed understanding of the life cycle is helpful to develop novel anti-influenza drugs and the development of influenza A virus high-yield cell lines for vaccine production. 

## 5. Conclusions

In this study, we found that KAP1 promotes the propagation of influenza A virus in human lung epithelial cells. KAP1 interacts with PB2 and NS1 proteins during the virus infection. KAP1 facilitates the early steps, promoting the viral proteins synthesis, maintaining the polymerase activity, and inhibiting IFN production in the influenza virus life cycle. Our results are of value to deeply understand the life cycle of the influenza virus and provide the novel target for the development of antiviral drugs.

## Supplementary Materials

The following supporting information can be downloaded at: https://www.mdpi.com/article/10.3390/v14040689/s1, Figure S1: Knockout of KAP1 in A549 cells in clone 23 significantly reduced the influenza A virus growth; Figure S2: KAP1 did not interact with PB1, PA, HA, M1, and M2 proteins during influenza A virus infection in A549 cells; Figure S3: KAP1 in not involved in the vRNP import and export in A549 cells during the virus infection; Figure S4: Deletion of KAP1 did not significantly affect the VLP formation and release in 549 cells.
