# Supplementary material for: KAP1 Positively Modulates Influenza A Virus Replication by Interacting with PB2 and NS1 Proteins in Human Lung Epithelial Cells
Source: Viruses. 2022 Mar 26;14(4):689. doi: 10.3390/v14040689 (PMC9025026; doi:10.3390/v14040689)
Supplement: Supplementary file 1 [file viruses-14-00689-s001.zip › viruses-1649297-supplementary.pdf]

Supplementary Information

A

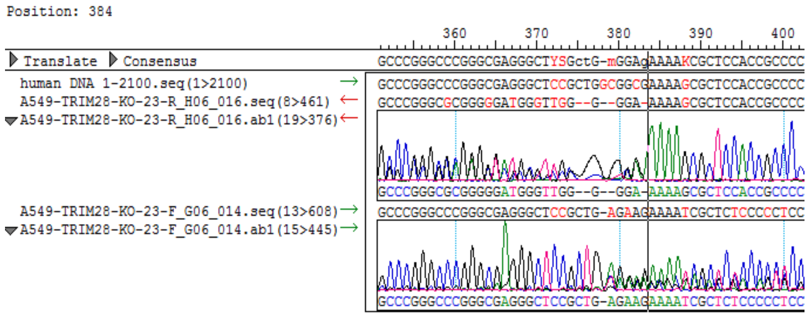

B

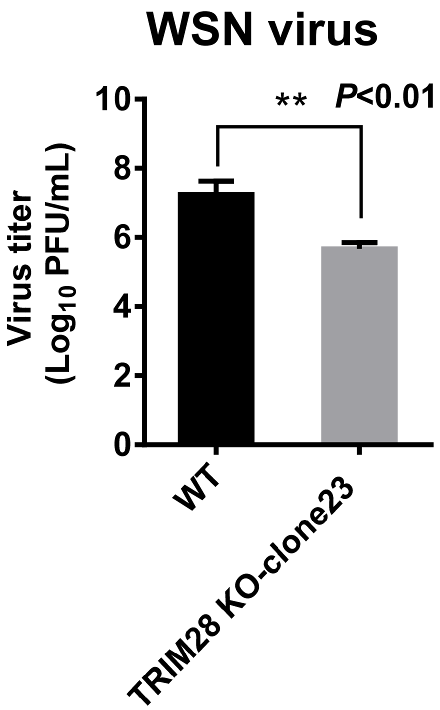

Figure S1. Knockout of KAP1 in A549 cells in clone 23 significantly reduced the influenza A virus growth. (A) Gene editing of KAP1 in clone 23 A549 cells was confirmed by Sanger sequencing.(B) The virus titer in clone 23 of KAP1 KO A549 cells was significant lower than that in WT A549 cells.

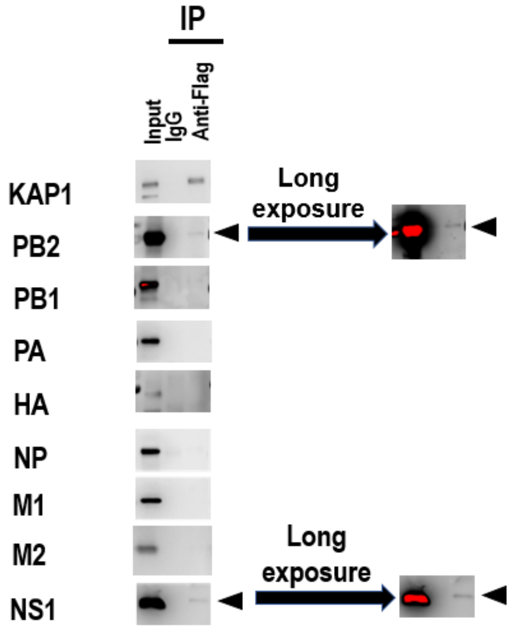

Figure S2. KAP1 did not interact with PB1, PA, HA, M1, and M2 proteins during influenza A virus infection in A549 cells. (A) WT or KAP1-FLAG overexpression A549 cells were infected with the WSN virus at an MOI of 10. The cell lysates were harvested at 12 hpi and immunoprecipitated (IP) by using an anti-FLAG antibody and Dynabeads. The products were subjected to Western blotting and detected with the indicated viral antibodies.

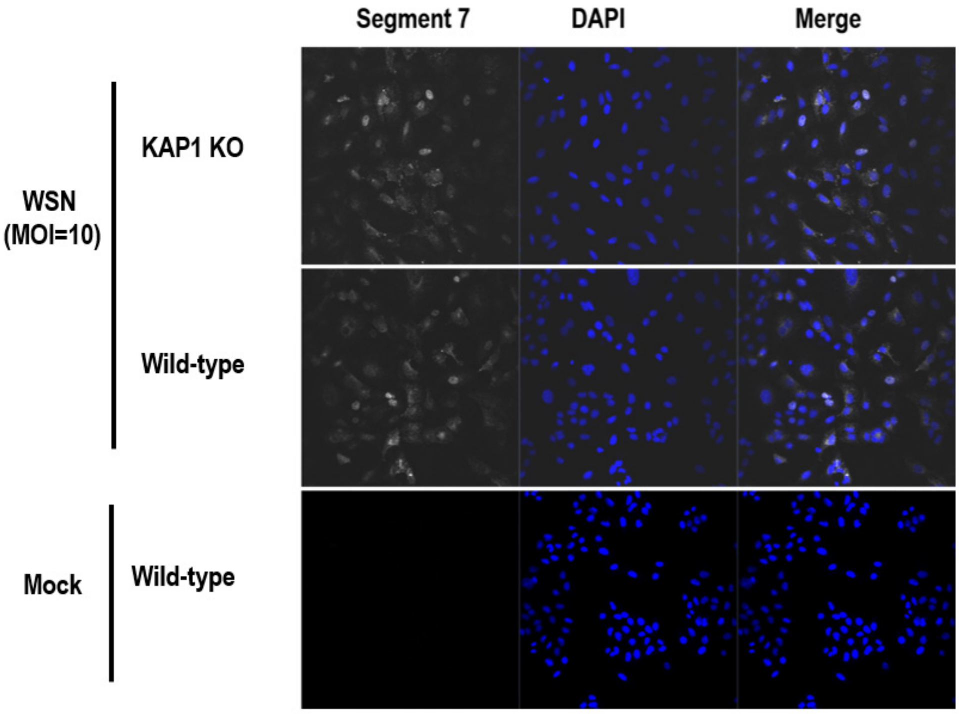

Figure S3. KAP1 in not involved in the vRNP import and export in A549 cells during the virus infection. WT or KAP1 KO A549 cells were infected with the WSN virus at an MOI of 10. Mock-infected A549 cells were used to be as a negative control. The infected cells were fixed at 12 h post-infection and then were stained with segment 7 vRNA probe and the nucleus were stained with Hoechst 33342. After the coverslips were mounted in ProLong Gold anti-fade mounting media. The cured samples were subjected to confocal microscopy examination.

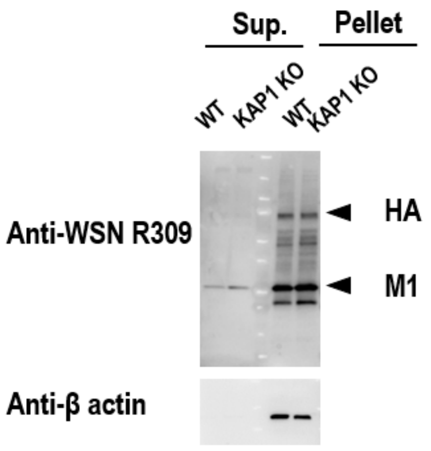

Figure S4. Deletion of KAP1 did not significantly affect the VLP formation and release in 549 cells.

The KAP1 KO and wild-type A549 cells were transfected with pCAGGS plasmids expressing HA, NA, and M1. After 48 h, the supernatants were collected and centrifugated by 30% sucrose at 40,000 rpm for 2 h at 4°C. For the transfected cells, 2×SDS sample buffer with DTT was added into each well and subjected them to Western blotting after sonication and heating. The viral HA, NP and M1 proteins were detected with R309 antibodies.
